# Invasive Group A Streptococcal Infection Following Nasal Self-Packing With Facial Tissue: A Case Report

**DOI:** 10.7759/cureus.92592

**Published:** 2025-09-17

**Authors:** Takeo Mori, Sadako Yoshizawa, Naoki Onda, Yamato Mifune, Fumio Sugo, Katsuhito Kashiwagi, Takahiro Sato, Tadashi Maeda, Norihiko Takemoto, Yosuke Sasaki

**Affiliations:** 1 Department of General Medicine and Emergency Care (Infectious Diseases), Toho University Omori Medical Center, Tokyo, JPN; 2 Department of Microbiology and Infectious Diseases, Toho University School of Medicine, Tokyo, JPN; 3 Department of Clinical Laboratory, Toho University Omori Medical Center, Tokyo, JPN; 4 Department of Laboratory Medicine, Toho University School of Medicine, Tokyo, JPN; 5 Department of Hematology and Oncology, Toho University Omori Medical Center, Tokyo, JPN; 6 Department of Cardiovascular Medicine, Toho University Omori Medical Center, Tokyo, JPN; 7 Department of Obstetrics and Gynecology, Toho University Omori Medical Center, Tokyo, JPN; 8 Department of Infectious Diseases, National Institute of Global Health and Medicine, Japan Institute for Health Security, Tokyo, JPN; 9 Department of General Medicine and Emergency Care, Toho University School of Medicine, Tokyo, JPN

**Keywords:** bacteremia, emm typing, immunocompromised host, multiple myeloma, nasal infection, streptococcus pyogenes

## Abstract

Invasive group A streptococcal infection (iGAS) can develop rapidly in immunocompromised individuals. We describe the case of a 66-year-old man with multiple myeloma who was admitted for chemotherapy and subsequently developed iGAS. He had been performing nasal self-packing with facial tissue for nasal discharge and hemorrhage. During hospitalization, he developed erythema, pain, and swelling around the nose and experienced a sudden onset of septic shock characterized by profound hypotension requiring norepinephrine infusion. *Streptococcus pyogenes* was detected in blood cultures, and treatment with beta-lactam antibiotics and clindamycin led to rapid improvement. Follow-up testing revealed that *emm* typing identified the strain as *emm49*. This case highlights that in immunocompromised individuals, trivial self-care like nasal self-packing can precipitate severe infections such as iGAS and shock, emphasizing the need for clinical vigilance.

## Introduction

*Streptococcus pyogenes* is a well-known causative pathogen of skin and soft tissue infections, such as erysipelas and cellulitis, and in some cases, it can lead to severe conditions, including necrotizing fasciitis and toxic shock syndrome (TSS) [1]. In particular, invasive group A streptococcal infections (iGAS) and streptococcal toxic shock syndrome (STSS), which are triggered by toxins produced by the bacterium, are known for their rapid progression and high mortality rates [1].

TSS has been reported to occur in women as a result of using menstrual products such as tampons [2]. Regardless of gender, there have been some reports of TSS caused by *S. pyogenes* and *Staphylococcus aureus* following nasal sinus surgery or nasal packing for epistaxis [3-5]. We report a case of iGAS in a patient with a history of multiple myeloma, who developed the infection after performing nasal self-packing with facial tissue for nasal discharge and hemorrhage. This case suggests that, particularly in immunocompromised patients, inserting facial tissue into the nasal cavity may serve as a potential risk factor for iGAS. We report this case to highlight important considerations for similar future cases.

Informed written consent was obtained from the patient for the open-access publication of this case report.

## Case presentation

A 66-year-old male patient presented to our hospital with complaints of back pain. Two months prior to the onset of disease, he experienced movement-related back pain, nasal discharge, and hemorrhage. He had been performing nasal self-packing with facial tissue once a day. One month prior to onset, he was referred to our hospital for further evaluation of his back pain. He had a medical history of hypertension, dyslipidemia, gastroesophageal reflux disease, and idiopathic contact dermatitis. His regular medications included nifedipine, azilsartan, bisoprolol, vonoprazan, sodium picosulfate, and a combination tablet of tramadol and acetaminophen. He had a smoking history of two packs per day for 46 years, but no history of alcohol consumption or overseas travel.

Following his initial visit to our hospital, laboratory testing and computed tomography revealed anemia, protein-albumin dissociation, and multiple osteolytic lesions. Based on these findings, he was subsequently diagnosed with stage III multiple myeloma according to the Revised International Staging System, identified as IgG-producing type. He was admitted for treatment seven days prior to disease onset. Three days before onset, he received chemotherapy with bortezomib, lenalidomide, and dexamethasone. During this time, he also continued nasal self-packing with facial tissue.

**Figure 1:**
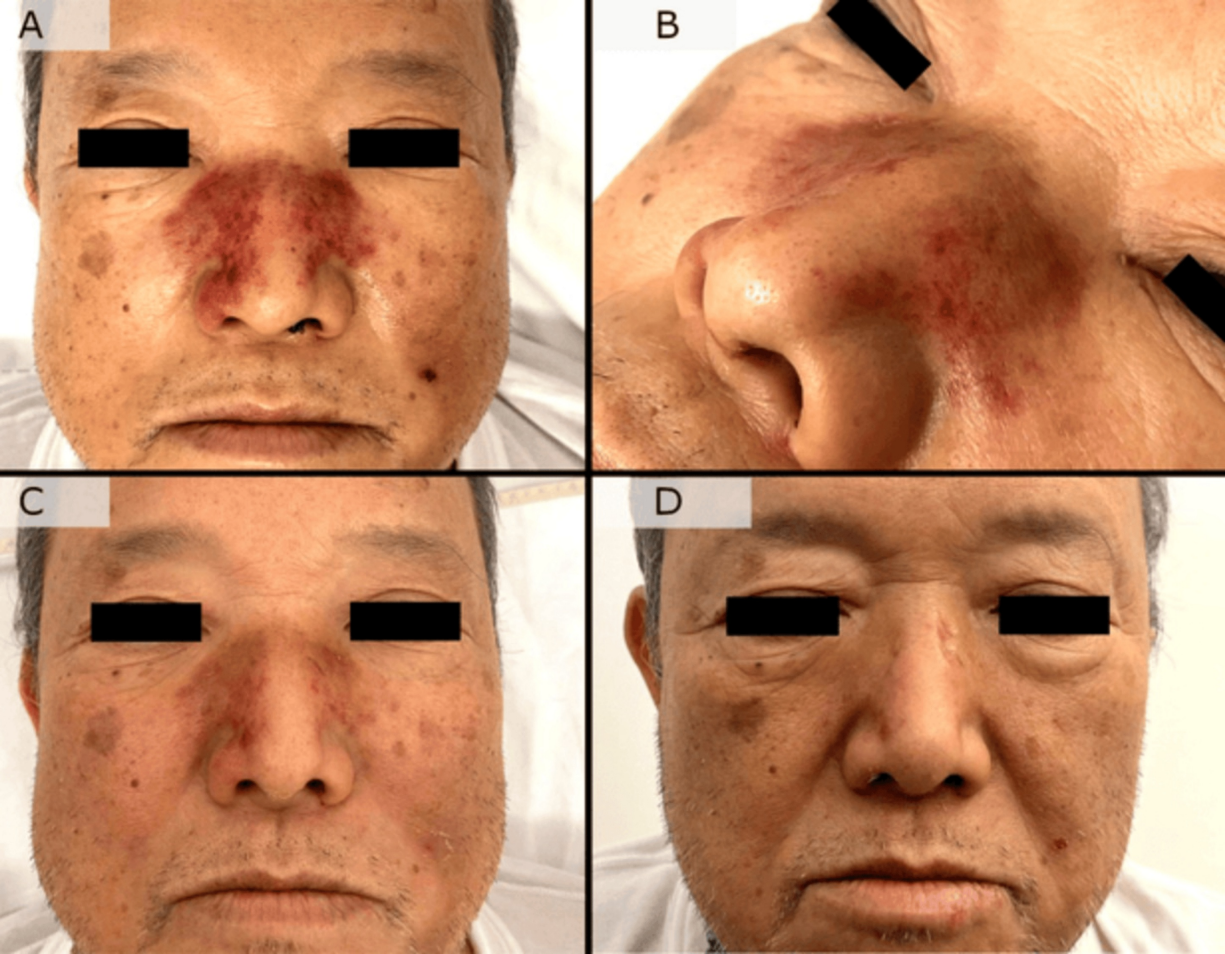
Clinical course of nasal erythema and swelling On the day of onset, physical examination revealed erythema and purpura with warmth, swelling, and tenderness over the nasal dorsum and alae (A). An enlarged view of the macroscopic photograph (B). On the second day after onset, the erythema and swelling persisted but showed slight improvement (C). On the fourth day after onset, local findings had disappeared (D)

On the day of onset, erythema with pain appeared around his nose, and a few hours later, he developed fever and shock. His vital signs were as follows: body temperature at 40.1°C, blood pressure at 68/47 mmHg, heart rate at 106 bpm, percutaneous oxygen saturation at 95% (room air), and respiratory rate at 24 breaths per minute, indicating shock and tachypnea. Physical examination revealed erythema and purpura with warmth, swelling, and tenderness over the nasal dorsum and alae (Figure 1). There were blood clots in the left nasal cavity. Bilateral tonsillar enlargement and erythema were observed, with white exudate on the left tonsil. No significant abnormalities were found in heart sounds, lung sounds, the abdomen, or the extremities.

Laboratory findings showed leukopenia, anemia, and thrombocytopenia, along with an elevated inflammatory response (C-reactive protein: 4.2 mg/dL). His renal function had deteriorated from 0.82 to 1.86 mg/dL the previous day. Procalcitonin was markedly elevated at 301.56 ng/mL. Immunoglobulin levels showed an increased IgG of 3,723 mg/dL, while IgA and IgM were suppressed. Other blood and urine test results are shown in Tables 1, 2. A chest X-ray showed pulmonary congestion and cardiac dilatation. Computed tomography revealed mucosal thickening and fluid retention in the maxillary and ethmoid sinuses and a fracture in the left nasal bone. Although differential diagnoses such as urinary tract infection, catheter-related bloodstream infection, and other causes of shock, including anaphylaxis, were considered, the combination of his clinical history and local nasal findings strongly suggested bacterial infection of the nasal cavity with septic shock. Blood cultures were performed, and treatment with meropenem and norepinephrine was initiated.

**Table 1:** Results of blood test on the day of onset with fever WBC: white blood cell, RBC: red blood cell, Hb: hemoglobin, Ht: hematocrit, MCV: mean corpuscular volume, MCH: mean corpuscular hemoglobin, MCHC: mean corpuscular hemoglobin concentration, Plt: platelet, UA: uric acid, UN: blood urea nitrogen, Cr: creatinine, AST: aspartate aminotransferase, ALT: alanine aminotransferase, LDH: lactate dehydrogenase, γ-GTP: gamma-glutamyl transpeptidase, CK: creatine kinase, HbA1c: hemoglobin A1c, CRP: C-reactive protein, PCT: procalcitonin, IgG: immunoglobulin G, IgA: immunoglobulin A, IgM: immunoglobulin M, PT: prothrombin time, PT-INR: prothrombin time-international normalized ratio, PT%: prothrombin activity (%), APTT: activated partial thromboplastin time, FDP: fibrin degradation product

Laboratory parameter	Observed value	Reference range
WBC (/μL)	3,200	3,300-8,600
Basophil (%)	0	0-2
Eosinophil (%)	0	1-9
Lymphocyte (%)	6	25-48
Meta-myelocyte (%)	1	0
Segment cell (%)	85	22-72
Band cell (%)	3	0-18
Monocyte (%)	5	2-12
RBC (×10^4^/μL)	211	435-555
Hb (g/dL)	8.8	13.7-16.8
Ht (%)	25.9	40.7-50.1
MCV (fL)	122.7	83.6-98.2
MCH (pg)	41.7	27.5-33.2
MCHC (%)	34	31.7-35.3
PLT (×10^4^/μL)	10.9	15.3-34.8
Na (mEq/L)	142	138-145
K (mEq/L)	3.6	3.6-4.8
Cl (mEq/L)	102	101-108
UA (mg/dL)	7.2	3.7-6.9
UN (mg/dL)	42	8-20
Cr (mg/dL)	1.86	0.65-1.07
AST (IU/L)	28	13-30
ALT (IU/L)	18	10-42
LDH (IU/L)	361	124-222
γ-GTP (IU/L)	52	13-64
CK (IU/L)	47	58-248
Glucose (mg/dL)	86	73-109
HbA1c (%)	5	4.9-6.0
CRP (mg/dL)	4.2	0.0-0.2
PCT (ng/mL)	301.56	<0.05
IgG (mg/dL)	3,723	861-1,747
IgA (mg/dL)	12	93-393
IgM (mg/dL)	<10	33-183
PT (seconds)	14.9	9.6-13.1
PT-INR	1.4	0.8-1.2
PT% (%)	58	70-130
APTT (seconds)	35.4	24.0-39.0
Fibrinogen (mg/dL)	364	200-400
FDP (μg/mL)	55.6	<5
D-dimer (μg/mL)	34.1	<1.0

**Table 2:** Results of arterial blood gas analysis and urine tests on the day of onset with fever PaO_2_: arterial oxygen partial pressure, PaCO_2_: arterial carbon dioxide partial pressure, cHCO_3_⁻: calculated bicarbonate, HPF: high-power field, WBC: white blood cell, RBC: red blood cell

Laboratory parameter	Observed value	Reference range
Arterial blood gas analysis (nasal cannula at 2 L/minute O₂)		
pH	7.410	7.38-7.46
PaO_2_ (mmHg)	77.1	74-108
PaCO_2_ (mmHg)	31	32-46
CHCO_3_^-^ (mEq/L)	19.3	22.5-26.9
Lactate (mmol/L)	1.5	0.5-1.6
Urinalysis		
pH	5	4.5-7.5
Protein	+	-
Sugar	-	-
Acetone	-	-
Nitrite	-	-
Sediment WBC (/HPF)	5-9	0-4
Sediment RBC (/HPF)	10-19	0-4

The next day, Gram-positive streptococci were detected in blood cultures. Matrix-assisted laser desorption/ionization (MALDI) time-of-flight mass spectrometry using MALDI Biotyper (Bruker Daltonics GmbH, Bremen, Germany) from the blood identified it as *S. pyogenes*. Because STSS was highly considered, clindamycin was added to the meropenem regimen. The treatment course was favorable, with local symptoms showing a tendency to resolve and become slightly less prominent by the second day of treatment, allowing discontinuation of norepinephrine. By the fourth day, local findings had disappeared (Figure 1).

The final identification of the bacteria was *S. pyogenes*, and the same pathogen was also isolated from the pharyngeal mucosa and the nasal cavity. *emm* gene analysis identified the strain harbors *emm49*. The results of antimicrobial susceptibility are shown in Table 3. Based on final identification and antimicrobial susceptibility results, the antibiotic regimen was changed to intravenous ampicillin and clindamycin. Once the patient recovered from shock and vital signs stabilized, administration of clindamycin was discontinued. The patient underwent 11 days of intravenous therapy and was discharged for personal reasons. Because of concerns about a relapse due to the short duration of therapy for bacteremia, he was given one week of oral amoxicillin. After a total of 18 days of treatment, there is no recurrence.

**Table 3:** Results of drug susceptibility testing for isolated S. pyogenes MIC: minimum inhibitory concentration

Antibiotics	MIC (μg/mL)
Penicillin G	≦0.06
Ampicillin	≦0.25
Sulbactam/ampicillin	≦0.25
Cefotiam	≦0.5
Ceftizoxime	≦0.25
Cefpirome	≦0.5
Cefditoren-pivoxil	≦0.12
Imipenem	≦0.12
Meropenem	≦0.12
Clarithromycin	≦0.12
Minocycline	≦0.25
Vancomycin	≦0.5
Teicoplanin	≦0.5
Tosufloxacin	≦0.5
Levofloxacin	1
Clindamycin	≦0.12

## Discussion

Patients with multiple myeloma are known to have an increased risk of infections due to impaired production of normal immunoglobulins [6]. In particular, secretory IgA has a role in mucosal infection defense, and IgG3 is involved in the initial immune response to the M protein of *S. pyogenes* [7,8]. This risk is particularly high in advanced cases and during the early stages of diagnosis [9]. Previous reports suggested that the incidence of infectious diseases can be up to seven times higher in these patients [10]. In 2023, a case of toxic shock-like syndrome caused by *Streptococcus agalactiae* of unknown entry site was reported in a patient with treatment-resistant multiple myeloma [11]. As in previous reports, the present case was observed at both the initial diagnosis stage and an advanced disease state, suggesting a possible association. Although this case did not meet the criteria for STSS as defined by the Centers for Disease Control and Prevention, the patient presented with rapidly developing shock, along with vital signs, respiratory failure, renal impairment, and disseminated intravascular coagulation, suggesting a case very close to STSS.

As seen in this patient, long-term retention of foreign bodies in the nasal cavity can stimulate local bacterial growth. A previous study reported the detection of Klebsiella spp., *Proteus mirabilis*, *S. aureus*, *Streptococcus pneumoniae*, and *Haemophilus influenzae* from packing materials used after hemorrhage and sinus surgery [12,13]. Cases of infective endocarditis and spondylitis caused by *S. aureus* following nasal septum surgery have also been reported [14]. Additionally, cases of TSS caused by *S. pyogenes* and *S. aureus* have been reported after nasal or sinus surgery, suggesting that nasal packing may be a risk factor for TSS [3-5]. In the present case, it is presumed that the enclosed environment created by facial tissue in the nasal cavity facilitated the colonization and local proliferation of *S. pyogenes*. A previous study involving 350 healthy volunteers aged 18 years or older in Poland reported a pharyngeal carriage rate of *S. pyogenes* of 4.9%, highlighting that, even among healthy adults, asymptomatic colonization can occur [15].

The isolated *S. pyogenes* strain was identified as *emm49*. The *emm* gene encodes the M protein of *S. pyogenes*, which is an important virulence factor involved in cell adhesion, resistance to phagocytosis, and inhibition of complement system activation [16]. The *emm49* strain has been increasingly reported in recent years in the United States, the United Kingdom, Canada, and Spain [17-19]. In an analysis of strains obtained from 526 STSS patients in Japan between 2019 and 2021, *emm49* accounted for 8.4% of the total, making it the fourth most common type following *emm1*, *emm89*, and *emm81* [20]. Its proportion increased from 3.4% in 2019 to 13.5% in 2022 [20]. It has also been shown that the *emm49* strains carrying a mutation in the *scpA* gene, which encodes the C5a peptidase protein involved in promoting local infections such as skin and soft tissue infections, are spread in Arizona [21,22]. Also, in our case, inflammation of the skin and soft tissue around the nose was observed. While the M1UK strain has drawn attention in cases of severe *S. pyogenes* infections, considering epidemiological data, it is also important to recognize the potential threat of the *emm49* strains [23].

This case highlights that the presence of a foreign body in the nasal cavity, such as facial tissue, particularly in immunocompromised patients, may serve as a trigger for severe bacteremia. Recognizing this risk emphasizes the importance of infection prevention education for immunocompromised patients. Additionally, with the increasing prevalence of highly virulent *S. pyogenes* strains, there is a concern that similar cases may become more frequent in the future.

## Conclusions

This case represents a rare clinical course of iGAS in a patient with multiple myeloma, and nasal self-packing with facial tissue was considered a possible trigger. The presence of a foreign body in the nasal cavity may have facilitated local bacterial colonization and proliferation and led to systemic infection. The isolated *S. pyogenes* strain was identified as *emm49*, a genotype that has been increasing in prevalence in recent years and is suggested to be associated with pathogenicity. Beyond its rarity, this case highlights key lessons for clinical practice: even seemingly trivial self-care behaviors can carry serious risks in immunocompromised individuals, and clinicians should maintain vigilance for iGAS when unusual local findings are present. Awareness of these risks may help reduce the likelihood of similar severe infections in the future.
